# The use of annual physical examinations among the elderly in rural China: a cross-sectional study

**DOI:** 10.1186/1472-6963-14-16

**Published:** 2014-01-14

**Authors:** Xi Sun, Yingchun Chen, Xuetao Tong, Zhanchun Feng, Li Wei, Donghua Zhou, Miaomiao Tian, Benyan Lv, Da Feng

**Affiliations:** 1 Medicine and Health Management School, Tongji Medical College, Huazhong University of Science and Technology, 13 Hangkong Rd., Wuhan, Hubei 430030, PRC; 2 Union Hospital, Tongji Medical College, Huazhong University of Science and Technology, 1227 Jiefang Rd, Wuhan, Hubei 430022, PRC; 3 Institute of Medical Information, Center for Health Policy and Management, Chinese Academy of Medical Sciences, 3 Yabao Rd, Chaoyang, Beijing 100020, PRC

**Keywords:** Rural, Annual physical examination, Health services use, Elderly, Chinese

## Abstract

**Background:**

Periodic physical examination is considered helpful in preventing illness and promoting health among the elderly. Limited information is available about the use of annual physical examinations among the elderly in rural areas, however. This research explores the distribution characteristics of annual physical examination use and its determinants among people aged 60 or over in rural China.

**Methods:**

A cross-sectional study was undertaken to estimate distribution characteristics of annual physical examination use and to collect data of sociodemographic characteristics, health knowledge level, and health communication channels. Participants were 1128 people aged 60 or over, randomly selected from four different provinces in the East, Mid-East, Mid-West, and West China. Logistic regression determined the predictors of annual physical examination use.

**Results:**

Participants were predominantly aged 60–79 (44.1%) and 70–79 (42.0%). A total of 716 (63.5%) participants underwent annual physical examinations. Those who reported acquiring health knowledge via bulletin boards and village doctors had a higher probability of using annual physical examinations (OR = 3.15 and 1.53). The probability for civil servants/retired having annual physical examinations was 2.16 times higher than for farmers. Those who had an average level of health knowledge had a higher probability of using annual physical examinations than those at the below-average level (odds ratio: 2.07).

**Conclusion:**

The government and public health institutions should assist farmers to acquire the habit of having annual physical examinations. Traditional channels, such as bulletin boards, should be used to deliver health information. Village doctors should be supported in delivering health information to the elderly in rural areas.

## Background

Periodic physical examination is considered an important and effective measure in the prevention of illness and promotion of health [1,2]. Moreover, it provides an opportunity for health status evaluation, preventive health consultation, and physician-patient relationship promotion [3]. In some situations, such as screening for cancer, periodic physical examination is very important [4,5].

Good health is essential for the elderly to maintain an independent lifestyle and make a contribution to their families and communities [6]. However, the elderly often suffer from diseases, especially chronic diseases such as hypertension, diabetes, and dyslipidemia, and these diseases are a heavy economic burden for the elderly [7]. Therefore, it is necessary and appropriate for the elderly to have periodic physical examinations [8].

Population aging in China is already happening; the proportion of those aged 60 or over increased by 3.82%, from 9.67% to 13.49%, between 1990 and 2010 [9]. Only 14.6% of rural Chinese adults aged 65 or above had an annual physical examination in 2008 [10]. In China, county hospitals, town hospitals, and village clinics compose the three-tier rural health service network. They are the principal providers of health care services to rural residents to maintain their health. Under the new health care reform scheme, annual physical examinations were to be provided free of charge to adults aged 65 or over in rural China by town hospitals from 2009. In some regions, the age requirement was lowered to 60 [11,12]. In the past 3 years, from 2009 to 2012, the take-up of annual physical examinations among people aged 60 or over increased rapidly, especially among people aged 65 or over [13,14].

Many studies have explored factors that affect the use of preventive health services, such as educational level, income, and self-rated living status [15]. The factor of economic barriers, in particular, has been mentioned frequently. People who have high income and private insurance are able to access preventive health services more easily [16-18]. Some studies [1,19-22] have also suggested that men are less likely to use preventive health services than women. Green and Pope (1999) mention that there are two views to explain why women use preventive health services more frequently. Some believe it is because women have higher morbidity rates, while others argue that it is because they are more sensitive to symptoms and care more about their health [19].

Age, income, and chronic diseases are also influencing factors in using community health services by elderly Chinese [23]. This means that those who are older, have a higher income, and who suffer with chronic diseases are more likely to use community health services. In other studies, elderly people who live alone, so-called empty nesters, use fewer community health services [24]. Health communication based on community can help the elderly to establish healthy behavior patterns and living habits, which can be effective in preventing and controlling the occurrence of various chronic diseases [25].

For the past 3 years, free annual physical examinations have been provided to Chinese adults aged 65 or over (60 or over in some regions) [11,12]. To our knowledge, there are almost no studies about the use of annual physical examination among adults aged 60 or above in rural China. As the predictors for usage of annual physical examinations among this group are unknown, this study examined the level and predictors for their availing of annual physical examinations. Specifically, the research questions are *(1) To what extent do adults aged 60 or over in rural China avail of the annual physical examination? (2) What are the potentially modifiable factors associated with availing of this service?*

## Methods

### Study population

**Figure 1:**
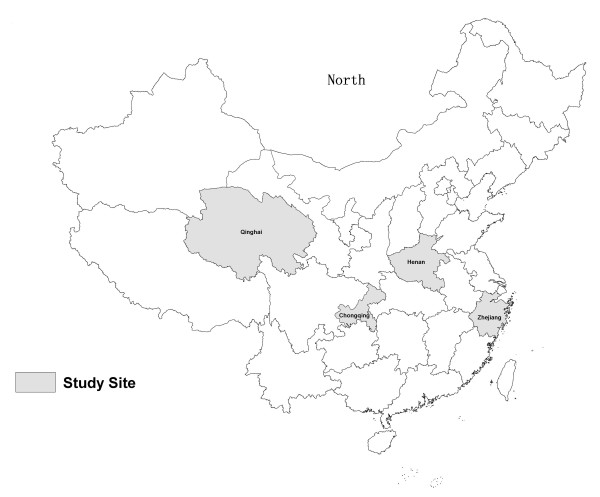
Distribution map of the study sites.

A cross-sectional survey study using stratified multi-stage sampling was conducted in July and August 2011. The sampling procedure involved five levels: province/municipality, county, township, village, and respondent. First, all the 31 provinces/municipalities in China were divided into four groups according to the Human Development Index [26], and one province/municipality was selected at random from each group. Zhejiang Province (in East China), Henan Province (in Mid-East China), Chongqing Municipality (in Mid-West China), and Qinghai Province (in West China) were chosen as the study sites (Figure 1). Second, each of the four selected study sites' counties were divided into three groups according to their economic development level, and one county was selected from each group as the representative county (4 × 3 = 12 counties selected). Third, the same procedure was repeated at the township level to select the representative townships (4 × 3 × 3 = 36 townships selected). Fourth, all the villages in each selected township were grouped into three categories according to their distance from the center of the town, and one village was selected randomly from each category (4 × 3 × 3 × 3 = 108 villages selected). Fifth, 11 or 12 rural adults aged 60 or over were selected at random in each selected village from the candidates listed in health records. A total of 1128 adults aged 60 or over were thus sampled from the 108 villages and completed the survey.

Face-to-face interviews were conducted by interviewers with each participant, and the study purpose was clearly explained to them. Students from the School of Medicine and Health Management of Tongji Medical College and staff members of local health care institutions were recruited and trained as interviewers. Survey items and response choices were read to participants who had difficulty reading because of illiteracy or poor vision.

This study was approved by the Ethics Committee of Tongji Medical College, Huazhong University of Science and Technology. Informed consent was obtained from all participants in the study.

### Questionnaire

All participants completed the Use of Annual Physical Examination Survey (Additional file 1). The questionnaire was delivered in simplified Chinese and contained closed-ended questions (true/false/don’t know format). The questionnaire consisted of three parts: 1) basic information; 2) health knowledge; and 3) means of acquiring health knowledge. The basic information comprised demographic and socioeconomic information. The demographic information collected comprised gender, age, and number of household members. The socioeconomic information collected comprised educational level, occupation, annual disposable household income, type of health insurance, and time required to reach township hospitals. Health knowledge items were developed by referring to the *2008 Chinese Citizens’ Health Literacy Survey*[10] and health brochures delivered to residents in rural China. On the basis of these materials, we identified 10 items that were easily understandable by the elderly for our questionnaire. The total number of correct answers was calculated as the overall health knowledge score. No points would be awarded for incorrect or missing answers or the answer “don’t know.” Participants who obtained a score at/or above eight points were considered to have an above-average level of health knowledge. Those who obtained a score of five points or less were considered to have a below-average level of health knowledge. Those who obtained a score of six or seven were considered to have an average level of health knowledge. Health communication channels comprised TV or radio broadcasts, the Internet, bulletin boards, friends or family members, and doctors.

### Statistical methods

Basic sociodemographic characteristics of the participants were summarized using descriptive statistics. Prior to conducting logistic regression analysis to identify the predictors of annual physical examination use, Chi-square and Fisher’s exact tests (whenever appropriate) were used to explore differences in value of the covariates between user and non-user groups of annual physical examinations to select potential predictors for the regression analysis. Logistic regression was then applied to examine the potential predictors of annual physical examination use. The dependent variable was whether (1) or not (0) each participant had annual physical examination. Independent variables included: occupation, number of household members, health knowledge level, means of acquiring health knowledge.

In this research, all data were entered twice into the EpiData 3.1, while data entry screens were used to rectify incorrect entries (e.g., logic and input errors). Statistical analysis was conducted using SPSS statistics 12. Statistical significance level was set at 0.05.

## Results

### Sociodemographic characteristics of participants

Slightly more than half (53.9%) of the participants were male, and most of them were farmers (78.6%). Participants were predominantly aged 60–79 (44.1%) or 70–79 (42.0%). More than 85% of them were at or below elementary educational level. Other demographic and socioeconomic characteristics are presented in Table 1.

**Table 1:** Sociodemographic characteristics of the elderly aged 60 or over in rural China

**Characteristics**	**Frequency (n = 1128)**	**Percentage (%)**
**Gender**		
Male	608	53.9
Female	520	46.1
**Age**		
60-69	497	44.1
70-79	474	42.0
80 or over	157	13.9
**Education level**		
Less than 6 years elementary study	610	54.1
Elementary	361	32.0
Middle school and above	157	13.9
Occupation^*^		
Farmer	887	78.6
Self-employed or Migrant worker	103	9.1
Civil servant or retired	138	12.2
**Type of health insurance**		
New cooperative medical system	1104	97.9
Non-new cooperative medical system	24	2.1
**Number of household members**		
1	97	8.6
2	278	24.6
3-4	388	34.4
5 or above	365	32.4
**Time required to reach township hospitals**		
Less than 10 min	858	76.1
10 min to 19 min	187	16.6
20 min and above	83	7.4
**Annul disposable household income**		
Less than CNY 10,000	441	39.1
CNY 10,000 to CNY 19,999	265	23.5
CNY 20,000 and above	422	37.4

*Self-employed means people who run a private small-scale business by themselves, such as food-shop owners, repair-shop owners and rice-noodles sellers. Migrant worker means the rural people who go into the cities to do an off-farm work but go back to their villages in harvest seasons and in Chinese traditional festivals.

Civil servant means people who are employed by the government, public sectors or enterprise held by the government. Retired means people who retired from these institutions.

### Annual physical examination use

Among the total 1128 participants, 63.5% (716) were users of annual physical examinations. Results of the bivariate analysis (Table 2) indicated significant differences between users and nonusers of annual physical examinations in some of the variables. The users tended to be civil servants or retired, belong to households of two or fewer members, have average or above-average levels of health knowledge, and acquire their health knowledge via TV or radio broadcasts, the Internet, bulletin boards, doctors, and family members and/or friends. No significant difference was reported between users and nonusers in gender, age, education level, type of health insurance, time required to reach township hospital, or annual disposable household income.

**Table 2:** Bivariate Correlates* of annual physical examination use among rural Chinese elderly aged 60 or over

**Potential predictor**	**User of annual physical examination**		**Nonusers of annual physical examination (n = 412)**		**χ**^**2**^	**p-value**
	**(n = 716)**					
	**N**	**%**	**N**	**%**		
**Occupation**						
Farmer	541	75.6	346	84	13.495	0.001
Self-employed or	69	9.6	34	8.3		
migrant worker						
Civil servant or retired	106	14.8	32	7.8		
**Number of household members**						
1	73	10.2	24	5.8	11.754	0.008
2	189	26.4	89	21.6		
3-4	237	33.1	151	36.7		
5 and above	217	30.3	148	35.9		
**Health knowledge level**						
Below average	403	56.3	286	69.4	19.793	<.001
Average	228	31.8	86	20.9		
Above average	85	11.9	40	9.7		
**Health communication channel**						
**TV or radio broadcast**						
No	276	38.5	240	58.3	40.914	<.001
Yes	440	61.5	172	41.7		
**Internet**						
No	633	88.4	393	95.4	15.493	<.001
Yes	83	11.6	19	4.6		
**Bulletin board**						
No	327	45.7	317	76.9	104.395	<.001
Yes	389	54.3	95	23.1		
**Doctor**						
No	144	20.1	166	40.3	53.435	<.001
Yes	572	79.9	246	59.7		
**Family member and friend**						
No	270	37.7	217	52.7	23.857	<.001
Yes	446	62.3	195	47.3		

*Only significant correlates are presented.

### Predictors of annual physical examination use among rural Chinese elderly

Because the bivariate effects of the predictors on the dependent variable were probably confounded by other factors, multivariate logistic regression analysis was further used to examine the predicting effect of each potential predictor identified in the bivariate analysis to adjust for the effects of other confounding variables. Four predictors—occupation, health knowledge level, and two health communication channels (doctors and bulletin boards)—were retained in the final logistic regression model to predict annual physical examination use among rural Chinese adults aged 60 or over (Table 3).Among all the significant predictors, the factor of health communication channel (bulletin boards) had the highest odds ratio. Those who reported acquiring their health knowledge via bulletin boards and village doctors had a higher probability of having annual physical examinations (odds ratio: 3.15 and 1.53). The probability for civil servants/retired was 2.16 times higher than for farmers. It is interesting that those who had an average level of health knowledge had a higher probability of using annual physical examinations than those at the below-average level (odds ratio: 2.07), but those who had an above-average level of health knowledge had almost the same probability as those with a below-average level (odds ratio: 0.95).

**Table 3:** Result of multivariate logistic regression analyses examining factors associated with annul physical examination use among rural Chinese elderly aged 60 or over

**Predictor**	**Reference category**	**B**	**OR**	**95% C.I.**		**p-value**
				**Lower**	**Upper**	
**Occupation**	Farmer					
Self-employed or		0.312	1.366	0.806	2.315	0.247
migrant worker						
Civil servant or		0.772	2.163	1.251	3.741	0.006
retired						
**Health knowledge level**	Below average					
Average		0.729	2.073	1.427	3.011	<.001
Above average		-0.054	0.948	0.565	1.591	0.840
**Health communication channel**						
Doctor	No	0.426	1.532	1.080	2.171	0.017
Bulletin board	No	1.147	3.149	2.219	4.467	<.001

## Discussion

In this study, we have not found any evidence for the impact of gender, type of health insurance, or annual disposable household income on the use of annual physical examinations by adults aged 60 or over in rural China. This result differed from those of some previous investigations [19,27,28], which examined factors predicting the use of other preventive or medical services. It is generally believed that females suffer from more diseases and are more sensitive to their health status. Moreover, the effects of economic barriers to preventive or medical services are common but were not identified as predictors in this study. This could be due to the wide coverage of the New Cooperative Medical System (NCMS) [29], which increased the financing standard from CNY30 to CNY300 per person and the personal payment standard from CNY10 to CNY60 per person [29,30]. To some extent, the NCMS has reduced the burden of disease, especially for adults in rural areas. In addition, annual physical examinations have been provided free of charge to the elderly in rural areas since 2009.

According to previous studies, higher occupational levels may contribute to an understanding of healthy behaviors [31]. This study showed that, compared with civil servants/retired, farmers were less likely to have annual physical examinations. There are two possible reasons. First, civil servants (either on the job or retired) are used to having annual physical examinations. A free medical insurance system (since the 1950s) and a medical insurance system for urban employees (since the 1980s) have been in place for many years, providing health insurance for civil servants employed by the government or in the public sector. Civil servants only have to pay a small part of the cost to receive an annual physical examination. By contrast, free annual physical examination services were unavailable to farmers until the healthcare reform of 2008, so they are not in the habit of using such services. Second, civil servants’ work units remind them when their annual physical examination is due and sometimes even arrange it for them. For civil servants who do not avail of the service, their units will remind them repeatedly. Farmers, by contrast, do not have scheduled physical examinations because they are busy farming. Although public health institutions have an obligation to remind them to have their annual physical examination, only one reminder is given.

According to the Knowledge, Attitude, Belief, and Practice (KABP) Model, health knowledge is the foundation of healthy attitudes, beliefs, and practices. People who have more health knowledge are more likely to behave healthily. In this study, we found that people who had an average level of health knowledge were more likely to avail of annual physical examinations, but having a level of health knowledge either below or above the average did not increase the likelihood of having an annual physical examination. It is reasonable to expect that those with a below-average level of health knowledge would be less likely to have an annual physical examination, but it is surprising that those with an above-average level were no more likely to have an annual physical examination. This may be explained by the fact that the free annual physical examination provided by the government only covers basic items. Those who have an above-average level of health knowledge may not believe in the value of such a basic examination. They may prefer to self-assess their health status on the basis of their health knowledge or to pay for a more detailed examination.

Health communication is essential to promoting and protecting the health of the public and is now being regarded as a vital core component of public health [32]. In general, the main objective of health communication is to influence individuals and communities. In rural Chinese regions, health communication channels include the interpersonal (e.g., doctors and family members or friends), mass media (e.g., TV or radio broadcasting), and the community-specific (e.g., local bulletin boards [25,33]). In this study, we found that the most popular and effective health communication channels are bulletin boards and village doctors.

Bulletin boards are very popular in rural China. They are set up and managed by village committees, and they have public credibility because they represent the public and government. All the important information about a village, such as election dates, natural disaster warnings, and physical examination times, will be published on its bulletin boards. Generally, the bulletin boards are set up beside the village committee’s workplace or in the gateway of the village so that they are convenient for villagers. It is believed to be an effective means of communicating health information because health communication channels that can reach a large proportion of the target population many times within a given period are more effective than those that do not [34,35].

Interpersonal communication is very important. This includes communication between the healthcare provider and the patient but also between the patient and others, especially other patients. Communication between healthcare providers and patients may affect the receipt of health services [36,37]. There are also a number of known barriers, such as language [27,28], gender [38], and disability [39], that can interfere with healthcare communication between providers and patients. Trust in physicians is also associated with the use of preventive health services; it is more important than trust in medical research and health information sources [40]. In particular, Chinese village doctors are indigenous and trusted by local villagers. They usually exhort villagers to avail of the annual physical examination. Communication between villagers is also very important [27,41], however, and health information from other patients is more easily trusted. That it was not shown to be a predictor in this study may be because of interference in provider-patient communication.

Mass media health campaigns have been an important strategy for health promotion and disease prevention. They are often more cost-effective than interpersonal channels because a very large audience can be reached after the initial investment in message production [42,43]. The public can receive various kinds of health knowledge from mass media because a large volume of health information is available to them. Adults in rural areas often watch or listen to health programs on TV or radio, the most popular mass media in rural China. In this study, however, the acquisition of information this way was not found to be a significant predictor of use of annual physical examination. This may be because there is almost no health information about the annual physical examination available via the mass media and because health programs are not tailored for the elderly in rural areas.

One of the limitations to this study is that some predictors found by other researchers (e.g., survey participants’ health status) were not included. Another research limitation is the use of closed-ended questions in the health knowledge test, which may have allowed participants to guess the correct answer. In addition, we assumed that the demographic and socioeconomic factors selected for this study, together with the selected health communication channels, are likely to be the main factors of influence for annual physical examination use among rural adults aged 60 or over in China, so other possible predictors were not considered.

## Conclusion

This study highlighted the relationship between health communication channels and annual physical examination use among adults aged 60 or more in rural China, as well as the predictors of the usage patterns of annual physical examination. The results emphasized the importance of two health communication channels, namely village doctors and bulletin boards, which are the most popular and trusted channels in rural China. The results also revealed the disparity among the adults of different occupations and levels of health knowledge.

On the basis of our findings and discussions, three recommendations can be given. First, the government and public health institutions should give special attention to farmers and help them to acquire the habit of having an annual physical examination. Second, we should pay more attention to the use of traditional channels of communication, such as bulletin boards, to deliver health information because they are visible, accessible, and cost-effective. Third, village doctors should be supported in delivering health information to the elderly in rural areas because they are trusted figures.

## Abbreviations

NCMS: New cooperative medical system.

## Competing interests

The authors declare that they have no competing interests.

## Authors’ contributions

Study concept and design: XS, YC and XT. Data collection: LW, DZ, MT, BL and DF. Statistical analysis: XS, YC and XT. Analysis and interpretation of data: SX, YC and XT. Draft of the manuscript: XS. Critical revision of the manuscript for important intellectual content: XS, YC, ZF, and XT. Administrative, technical, and material support: ZF and XS. Study supervision: ZF. All authors read and approved the final manuscript.

## Pre-publication history

The pre-publication history for this paper can be accessed here:

http://www.biomedcentral.com/1472-6963/14/16/prepub

## Supplementary Material

Additional file 1 **Questionnaire for annual physical examination use among the elderly in rural China.** Part A: Socio-demographic Characteristics. Part B: Health knowledge. Part C: Health communication channels. Part D: Use of annual physical examination. Click here for file
