# Supplementary material for: The use of annual physical examinations among the elderly in rural China: a cross-sectional study
Source: BMC Health Serv Res. 2014 Jan 14;14:16. doi: 10.1186/1472-6963-14-16 (PMC3925351; doi:10.1186/1472-6963-14-16)
Supplement: Additional file 1 — Questionnaire for annual physical examination use among the elderly in rural China. Part A: Socio-demographic Characteristics. Part B: Health knowledge. Part C: Health communication channels. Part D: Use of annual physical examination. [file 1472-6963-14-16-S1.doc]

**Questionnaire for annual physical examination use among the elderly in rural China**

**Part A: Socio-demographic Characteristics.**

A1、Date of birth: Year Month.

A2、Gender: (1) Male (2) Female

A3、Educational level: (1) No education (2) Elementary School (3) Junior Middle School (4) Senior Middle School (5) College or University and above

A4、Occupation: (1) Civil Servant (2) Farmer (3) Migrant Worker (4) Self-employed (5) Retired

A5、Health insurance type: (1) New Cooperative Medical Insurance (2) Non-New Cooperative Medical Insurance

A6、Number of household members in your family:

A7、Traveling time from your home to the nearest health institute: (1) Less than 10 mins (2) Less than 20 mins (3) 20 mins or above

A8、Your disposable household income in 2010.

(1) CNY5, 000 or below (2) CNY5, 001 - 10,000 (3) CNY10, 001- 15,000 (4) CNY15, 001- 20,000 (5) CNY20, 001 or above

**Part B: Health Knowledge.**

B1、A healthy person is neither overweighed nor underweighted, is able to eat and sleep, and does not have any sickness. Is it right?

(1) Yes; (2) No; (3) I don’t know

B2、Second-hand smoke (passive smoking) is not harmful to our health. Is it right?

(1) Yes; (2) No; (3) I don’t know

B3、Overweighed people are more likely to have diabetes compared with other people. Is it right?

(1) Yes; (2) No; (3) I don’t know

B4、Child vaccination is for preventing malnutrition. Is it right?

(1) Yes; (2) No; (3) I don’t know

B5、Anemia is related to the lack of iron in our body. Is it right?

(1) Yes; (2) No; (3) I don’t know

B6、Will having salty meals causes hypertension?

(1) Yes; (2) No; (3) I don’t know

B7、Will people be infected when sharing meals with Hepatitis B patients?

(1) Yes; (2) No; (3) I don’t know

B8、Will drinking too much alcohol lead to liver damaged?

(1) Yes; (2) No; (3) I don’t know

B9、It is safe to eat recently harvested fruits and vegetables after rubbing them with your hands. Is it right?

(1) Yes; (2) No; (3) I don’t know

B10、Dial 119 when you need emergency medical services. Is it right?

(1) Yes; (2) No; (3) I don’t know

**Part C: Health Communication Channels.**

C1、Did you receive health knowledge from TVs or radio broadcasts?

(1) Yes; (2) No

C2、Did you receive health knowledge from Internet (mobile phone or computer)?

(1) Yes; (2) No

C3、Did you receive health knowledge from doctors?

(1) Yes; (2) No

C4、Did you receive health knowledge from bulletin boards?

(1) Yes; (2) No

C5、Did you receive health knowledge from family members, neighbors, or friends?

(1) Yes; (2) No

**Part D: Use of Annual Physical Examination.**

D1、Did you receive free physical examination in 2010 (excluding physical examination conducted because of illness)?

(1) Yes; (2) No
